# Development and psychometric properties of a tool to assess Media Health Literacy (MeHLit)

**DOI:** 10.1186/s12889-022-14221-6

**Published:** 2022-10-01

**Authors:** Mahsa Nazarnia, Fatemeh Zarei, Nasrin Rozbahani

**Affiliations:** 1 Department of Health Education & Health Promotion, Faculty of Medical Sciences, Tarbiat Modares University (TMU), Tehran, Iran; 2 Department of Health Education & Health Promotion, Faculty of Medical Sciences, Tarbiat Modares University (TMU), P.O. Box: 14115-331, Tehran, Iran; 3 Department of Health Education & Health Promotion, School of health, Arak University of Medical Sciences, Arak, Iran

**Keywords:** Media Literacy, Health, Media Health Literacy

## Abstract

**Background:**

Media play an important role in shaping and changing the attitudes, thoughts, and behaviors of their audiences regarding health issues. Therefore, there is a need to explore and identify media health literacy concepts and domains.

**Methods:**

This is a cross-sectional study to design and psychometry an instrument to assess Media Health Literacy (MeHLit) from June to Septemper 2021. Relevant literature was reviewed to identify an item pool, and an expert panel was convened to choose items that might be included in the scale. The validity of the questionnaire was assessed through face validity (qualitative and quantitative),content validity (qualitative and quantitative) and construct validity (exploratory and confirmatory factor analysis) in 213 adults. Internal consistency of the scale was assessed by Cronbach’s alpha.

**Results:**

The content validity and reliability were demonstrated by CVR = 0.87 and CVI = 0.93, Cronbach’s α = 0.91. Exploratory factor analysis showed 5 factors including “Goal appraisal skill”; “Content appraisal skill” “Implicit message appraisal skill”; “Visual Comprehension skill”; “Audience appraisal skill”; which explained 60.25 of the variance.

**Conclusion:**

MeHLit is a valid and reliable questionnaire, with 21-item and 5 domains to assess media health literacy. To replicate the results obtained here, this measurement should be translated and maintained in other settings.

## Introduction

Nowadays, media play an important role in shaping and changing the attitudes, thoughts and behaviors of their audiences, therefore, the concept of media literacy has become one of the most important issues. Media literacy is one of the most important skills of the twenty-first century [1] defined as the capacity to access, analyse, examine and create messages throughout plenty of contexts [2] and a media literate person learns to use critical lenses both as consumers of media messages and as producers of their own messages [3] by decoding, evaluating, analyzing, and producing both print and electronic media [4] in all types of media as radio, television, cinema, music, magazines and internet on individuals and communities [5]. The European Charter of Media Literacy describes the seven main skills that media literate people should have as follows: “Effective use of media, access and informed decision-making about media content, understanding media content creation, analyzing media techniques and messages, using media for communication, abstaining from harmful media content and services, and using media for democratic and civil rights [6]. According to Media Literacy Index (MLI) in 2021 the highest MLI was reported in some European countries such as Finland, Denmark, Estonia, Sweden and Ireland [7]. On the contrary, the lowest and average MLI belonged to some Asian countries such as Turkey [7], Indonesia [8], and Iran [8–11] respectively.

Common literature in this field has considered media literacy as a predictive component of health literacy [9, 12] and has reported the positive effect of media literacy education on improving healthy lifestyles and promoting health [9] through identifying correct and incorrect information in social networks [13], infodemic and rumour management [14, 15], facilitating the search for information about disease prevention [16, 17] and recognizing the symptoms of diseases, recognizing physical and behavioral abnormalities [18].

Media Health Literacy (MHL) [19] builds on the integration of Health Literacy and Media Literacy [2]. These two interwoven concepts are noticeable in considering the scope and importance of eHealth literacy. The idea of MHL is beyond seeking health-related information through the media. However, it also considers implicit and explicit mass media content that may be health-promoting or unhealthy, usually produced by commercial entities or the healthcare system. In line with this, studies have shown that the main reason for spreading misinformation about diseases is poor medical advice social networks have been considered a key way to transmit this misleading medical information and enter into the community discourse. Also, mass media and elites in society have an effective role in socialization and changing attitudes and creating health-related behaviors. Therefore, understanding the formation of correct and incorrect attitudes and beliefs about health issues on the media platform emphasizes the disadvantage of equipping media literacy [20].

One of the most important principles in conducting any research is the availability of specific, valid and reliable tools for data collection [21]. Although many tools have been designed and psychometrically evaluated to measure the relationship between health literacy and media [22, 23] a review of the literature showed that there is no suitable tool for investigating media health literacy and its measurable domains are not yet fully understood. It seems that the scientific literature needs more research to identify its relation to health scope. Given MHL is a hybrid and unique concept measuring it as a general concept also needs a reliable, relevant and specific tool that to be reflected on its domains. Therefore, the present study was conducted with the aim of developing psychometric properties of a tool to assess the Media Health Literacy of Iranian adults.

## Method

### Design and sample

This is a cross-sectional study to design and psychometry an instrument to assess Media Health Literacy (MeHLit) in Iran. Participants were required with convenience samples through virtual social networks to complete an online questionnaire. Individuals were asked to participate in order to help rumour and infodemic management will occur in pandemics such as COVID-19. Data collection was conducted on Iranian adults from July to December 2021. The sample size was calculated based on an assumption of having at least 5–10 participants per item, with a 10% drop rate for incomplete questionnaires. Inclusion criteria were: being Iranian, ability to read and write Persian, age ≥ 18 years old, and registered in at least one of the popular online social networks such as WhatsApp, Instagram, and Telegram or its Iranian equivalent (e.g., Soroush, and Eitta). There were no other exclusion criteria for participation. Participation was voluntary and all participants had the right to discontinue the study at any time. The survey was conducted anonymously and participants’ contact information was kept confidential. The study was approved by the ethical research committee of Tarbiat Modares University (IR.MODARES.REC.1400.234).

### Scale development

To develop MeHLit two main following stages had been done: 1) Development of the questionnaire, and 2) Psychrometric properties of the questionnaire.

#### Development of the questionnaire

##### Conceptual framework and item generation

Media Health Literacy (MHL) [19] builds on the integration of Health Literacy and Media Literacy (ML) [2]. These are two concepts essential to understanding the scope and importance of eHealth literacy. The concept of media health literacy is unique in that it does more than just provide health advice considering information conveyed through the media. However, it should be also considered the implicit and explicit mass media content that may be health-promoting or unhealthy, usually produced by commercial entities or the healthcare system. Item generation was carried out based on a theology of Nutbeam on health literacy [24], and media health literacy conceptualization as a continuum of the following ranges: (1) the ability to identify health-related content (explicit and/or implicit) in the various types of media; (2) recognize the impact on health behavior; (3) critically analyzing content comparable to critical health literacy; (4) expressing intentions to respond through actions measured by personal health behavior or advocacy comparable to interactive health literacy [25]. The main components of the definition were contained in the scale. An extensive review was performed on the related literature. These led to the explanation of the concept of Media Health Literacy. Then, using the extracted concept, the practical definitions of the domains of Media Health Literacy were extracted.

##### Contextual item generation based on litreture review

Items of the questionnaire were also generated through the deductive-inductive approach. This approach helps to find appropriate items-based literature review on the dimensional structure and wording of the ML, combining the common keywords in ML and health. Accordingly, the initial items will answer five main questions in Media Health Literacy [26] when the audience comes across a health-related message in media. These consists of 1. Who created this message?; 2. What techniques are used to attract my attention?; 3. How might different people understand this message differently from me?; 4. What lifestyles, values, and points of view are represented in or omitted from this message?; and 5. Why was this message sent?.

At the end of this step a preliminary 30-item version of the questionnaire was ready to go through the psychometric stages.

#### Psychrometric properties of the questionnaire

For the psychometric properties of the questionnaire quantitative and qualitative face validity, quantitative and qualitative content validity, construct validity and reliability of MeHLit were assessed.

##### Face validity assessment

The face validity assesses whether a tool appears to measure what it’s supposed to measure [27]. The face validity was conducted in both qualitative and quantitative ways. In qualitative evaluation, the preliminary draft of MeHLit was assessed by 30 individuals who were similar to the target group. These participants assessed the difficulty, generality, and ambiguity of the items. The item’s impact scores were calculated to assess the face validity quantitatively. In this phase, the above participants rated each item using a 5-point Likert scale ranging from completely important to not at all important and gave a 5 to 1 rating. The following formula was used to calculate the item impact score: Item impact score = Frequency (percentage) × Importance. The items with an impact score of more than 1.5 were appropriate and maintained for the next stages [28].

##### Content validity assessment

Content validity assesses the instrument’s relevance, clarity, simplicity, and completeness [29]. The content validity of the MeHLit was tested qualitatively and quantitatively. In the qualitative content validity assessment, 10 key experts including; 2 health education specialists, 3 communication specialists, 3 media specialists, and 2 psychologists from a variety of Iranian public universities were asked to comment on the items in terms of wording, grammar, location in the scale, choice of vocabulary, appropriateness,and scoring [30]. In the quantitative content validity assessment, the content validity ratio (CVR) and the content validity index (CVI) were calculated. The content validity ratio was assessed by 10 experts. The experts determined the content validity ratio of each item with the criteria of “necessary”, “not necessary but useful” and “not necessary”. CVR was calculated through the following formula.

$$\mathrm{CVR}=\frac{\mathrm{N}e\frac{\mathrm{N}}{2}}{\frac{\mathrm{n}}{2}}$$

where *n*_*E*_ stands for the number of experts who have selected the option “essential” and *N* is the total number of experts. According to Lawshe’s table [31], the CVR higher than 0.62 for 10 individuals indicate the necessity of the item at a statistically significant level (*P* = 0.05). The Content Validity Index (CVI) was evaluated by the same 10 experts using a 4-point Likert scale that rated questionnaire items based on ‘simplicity’, ‘relevance’ and ‘clarity’ based on Waltz & Bausell’s content validity index [31]. CVI was calculated according to the following formula:

$$\mathrm{CVI}=\frac{\mathrm{Number}\ \mathrm{of}\ \mathrm{raters}\ \mathrm{chosing}\ \mathrm{points}3\ \mathrm{and}4}{\mathrm{Total}\ \mathrm{number}\ \mathrm{of}\ \mathrm{raters}}$$

*A C*VI score of 0.78 and above is considered acceptable [30].

##### Construct validity

To fine-tune the content of the questionnaire and assure the most parsimonious representation of the underlying components [32]. The Exploratory factor analysis (EFA) and Confirmatory factor analysis (CFA) method was used to assess the construct validity of MeHLit.

#### Exploratory factor analysis (EFA)

EFA was carried out through a cross-sectional study. Prichta et al. (2013) stated that the required number of respondents for EFA is 3–10 per item or 100–200 total respondents [32]. Therefore, 213 Iranian adults with experience in using media were recruited to complete the online questionnaires received from several popular online social networks such as WhatsApp, Instagram, and Telegram or its Iranian equivalent (e.g., Soroush, and Eitta). Data collection was done from June to September 2021, using convenience sampling. The tool for data collection was MeHLit following face and content validity assessment. No questionnaires were excluded from data entry from a total of 213 completed questionnaires. EFA was performed by the principal components method with varimax rotation and using SPSS version 22, and the indices used were the Kaiser-Meir-Olkin (KMO) index and Bartlett’s test of sphericity. KMO index indicates sampling adequacy and sufficient sample size to perform factor analysis. The value of this index is between zero and one, and the acceptable value for KMO is more than 0.5. Bartlett’s sphericity test was used to ensure the appropriateness of the data, which measures the significance of the data analysis and was considered at a significance level of 0.95. Three key indicators of eigenvalues, the ratio of explained variance, and scree plot were used to examine the amount and nature of MeHLit questionnaire factors. For each component, the item with a factor load of 0.4 and above was kept.

#### Confirmatory factor analysis (CFA)

Confirmatory Factor Analysis (CFA) is a statistical technique used to examine the factor structure of an observed variable [33]. Therefore, CFA was used to assess the MeHLit multi-dimensionality hypothesis using AMOS Software 24. The used indices were χ 2 whose insignificant amount indicates theoretical fitness with the data, the ratio of χ 2 to the degree of freedom in which the amount lower than 3 is preferred, and comparative fit index (CFI), the goodness of fit index (GFI), normed fit index (NFI) whose amounts higher than 0.9 were favorable for all these items. Regarding root mean square error of approximation (RMSEA) the amounts lower than 0.05 were very good and 0.08 were acceptable [34].

### Reliability assessment

Internal consistency, as one of Cronbach’s alpha reliability indexes was calculated in this study. This index’s satisfactory level was defined as being equal to or greater than 0.70 [35].

**Fig. 1:**
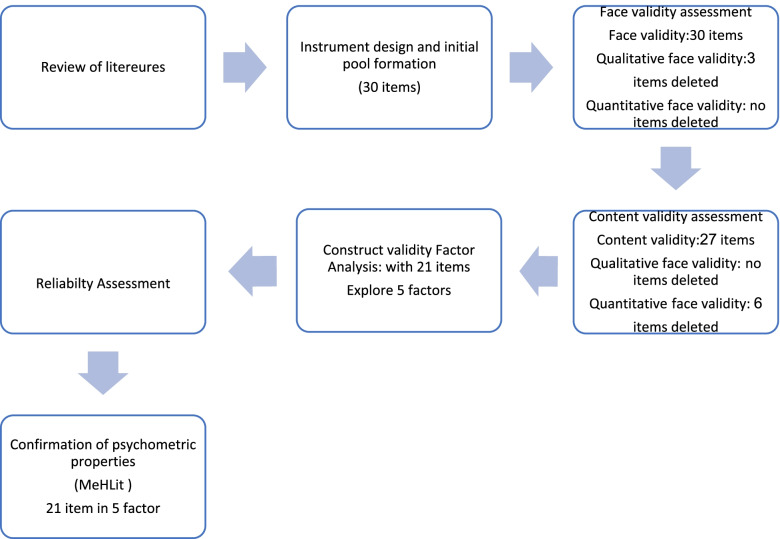
A summary of steps for designing and assessment of psychometric properties of MeHLit is presented in

A summary of steps for designing and assessment of psychometric properties of MeHLit is presented in Fig. 1.

### Description of the questionnaire: scaling and scoring

The current questionnaire consists of two parts;

- The general questions related to demographic issues include:
  age, gender, educational status, marital status, job status
- The main questions related to MeHLit

This part of the questionnaire aimed to assess Media Health Literacy regarding health messages. A 21-item questionnaire was finalized with five-point Likert response items in five domains; Goal appraisal skill, Content appraisal skill, Implicit me aning appraisal skill, Audience appraisal skill, and Visual Comprehension skill. The response to each item ranged from never (0), rarely (1), sometimes (2), most of the time (3), and always (4) The scoring ranges from 0 to 84, where the higher the score, person the more significant media literacy has to understand messages related to health issues.

## Result

The findings are presented in two parts:1) assessing the psychometric properties of the MeHLit; and 2) demographic results of participants.

### Assessment of psychometric properties of MeHLit

In the quantitative part, face, content, and construct validity and then the reliability of MeHLit was reported.

#### Face validity assessment

In the qualitative face validity assessment, 3 items were omitted for ambiguity and generality. In the quantitative face validity assessment, the importance of each item was measured and the items with an impact score of more than 1.5 were maintained. In this stage, all phrases received a score of more than 1.5. So, the 27 items remained.

#### Content validity assessment

In the qualitative content validity assessment, no items were deleted. In the quantitative content validity assessment, 6 items were deleted as they did not obtain acceptable CVI and CVR levels. The mean score of CVR was 0.87 (0.8–1). Also, the mean score of the CVI was 0.93 (0.82–1). Then, the questionnaire with 21 items entered the stage of construct validity assessment. Figure 1 shows the process of designing and assessing the psychometric assessment of MeHLit and the related changes to the questionnaire.

#### Construct validity assessment

**Fig. 2:**
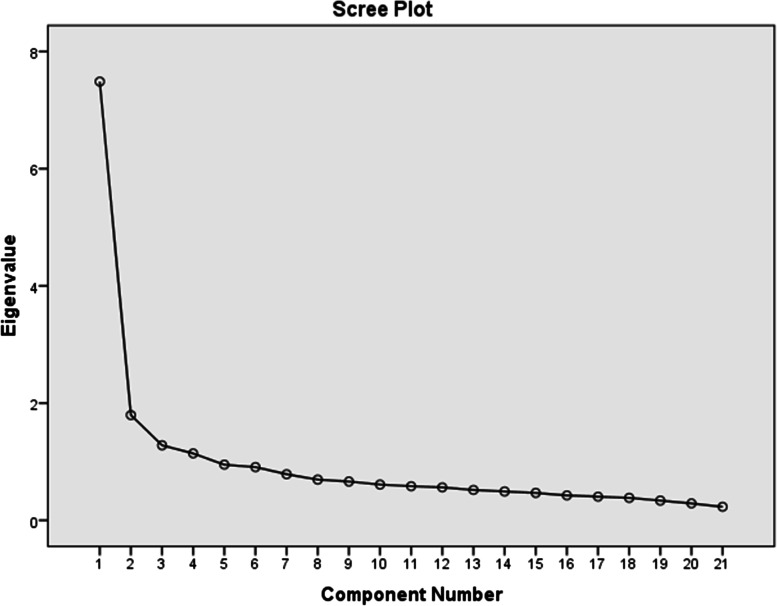
Scree plot of the exploratory factor analysis of MeHLit

**Fig. 3:**
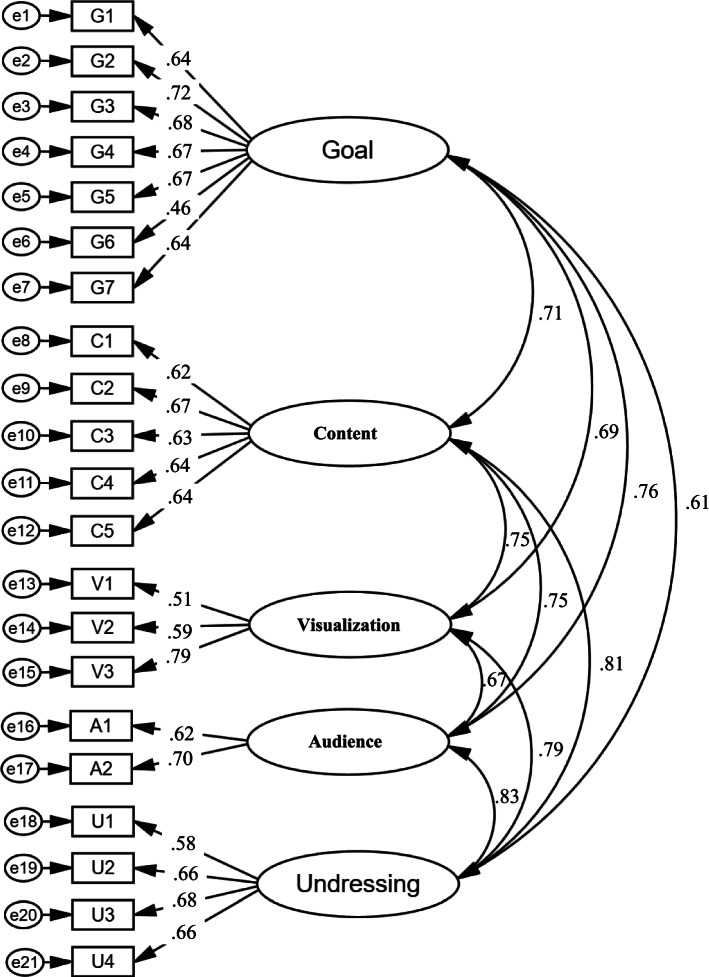
Confirmatory factor analysis model of MeHLit questionnaire

The sample size for this section of the study was considered 10 samples for each item. Thus, for 21 items, 213 individuals were recruited for the study. In total 213 individuals completed the questionnaire. Meanwhile, the calculated KMO index was 0.896 (× 2 = 1726.09,df = 210, *p* < .001) which showed the sample was adequate. In this matrix, variables that are highly correlated with each other are placed within a factor. Accordingly, Five factors that explained 60.25% of the cumulative variance of MeHLit were identified using the minimum eigenvalues of one (Table 1). After varimax rotation and considering the factor loading of at least 0.4, the items forming each factor were identified. The factors 1 to 5 are named as “Goal appraisal skill”; with 7 terms (explaining 35.65% of variance); “Content appraisal skill” with 5 items (explaining 8.55% of variance); “Implicit meaning appraisal skill” with 4 items (explaining 6.10% of variance); “Visual Comprehension skill” with 3 items (explaining 5.43% of variance); and “Audience appraisal skill” with 2items (explaining 4.52% of variance) respectively. A Scree plot was used to predict the number of factors. The scree plot suggested 5 factors that became the default for factor analysis (Fig. 2). In the next step, in order to confirm the structure obtained from the exploratory factor analysis, the confirmatory factor analysis was performed. RMSEA was 0.051 (less than 0.06) and the ratio of chi-square to the degree of freedom was 1.72 (less than 2). Other indicators also showed the optimal fit of the model (Table 2, Fig. 3).

**Table 1:** Exploratory analysis of MeHLit questionnaire

Items		Factors				
		Factor 1	Factor 2	Factor 3	Factor 4	Factor 5
Goal appraisal skill	When I come across a health-related message, I check for its purpose.	.750				
	When I come across a health-related message, I check for its educational point.	.739				
	When I come across a health-related message, I check for the explicit and direct meaning of the message.	.679				
	When I come across a health-related message, I check for what the message means to me.	.635				
	When I come across a health-related message, I check for that message from different aspects (application, utility, effectiveness).	.566				
	When I come across a health-related message, I think about deleting, keeping, or sending the message to someone else.	.539				
	When I come across a health-related message, I check for what thoughts and ideas it promotes.	.438				
Content appraisal skill	When I come across a health-related message, I think about the source of the message		.782			
	When I come across a health-related message, I think about the publisher of the message.		.715			
	When I come across a health-related message, I wonder if everyone understands this message the same way.		.609			
	When I come across a health-related message, I check its accuracy.		.532			
	When I come across a health-related message, I criticize it.		.525			
Implicit meaning appraisal skill	When I come across a health-related message, I pay attention to the negative and positive consequences of spreading it”.			.784		
	When I come across a health-related message, I note that who benefits from this message (financial, health, social benefits, etc.).			.784		
	When I come across a health-related message, I note that its implicit and hidden meaning (behind the scenes)			.563		
	When I come across a health-related message, I note who supported this message.			.465		
Visual Comprehension skill	When I come across a health-related message, I pay attention to audience attraction techniques (special effects such as color, light, sound, etc.).				.783	
	When I come across a health-related message, I pay attention to the method of distribution (virtual networks, mass media, print media)				.722	
	When I come across a health-related message, I think about the date of the message published.				.551	
Audience appraisal skill	When I come across a health-related message, I note that to who this message is for.					.786
	When I come across a health-related message, I note that to whether this message is right for me or not.					.415

**Table 2:** Summary results of confirmatory factor analysis fit statistics

RMSEA	IFI	NFI	AGFI	GFI	CFI	X^2^/df	*P* value
0.051	0.92	0.90	0.88	0.90	0.93	1.72	0.001

#### Reliability

The reliability of MeHLit was assessed by Cronbach α. The total Cronbach α. for MeHLit was 0.91. All domains of the questionnaire were positively and significantly correlated with each other (Table 3). After confirming the validity and reliability of the MeHLit, the questionnaire was finalized.

**Table 3:** Results of descriptive statistics, correlation and Cronbach’s alpha dimensions of the questionnaire

	Factors	Cronbach’s Alpha	Mean	Std. Deviation	1	2	3	4	5	6
1	Media health literacy	0.91	54.34	13.66	1					
2	Goal appraisal skill	0.78	20.34	4.70	.814^a^	1				
3	Content appraisal skill	0.82	12.61	4.12	.831^a^	.557^a^	1			
4	Implicit meaning appraisal skill	0.70	8.77	3.82	.800^a^	.468^a^	.584^a^	1		
5	Visual Comprehension skill	0.77	6.98	2.71	.730^a^	.480^a^	.521^a^	.532^a^	1	
6	Audience appraisal skill	0.70	5	1.89	.722^a^	.546^a^	.510^a^	.557^a^	.432	1

^a^Correlation is significant at the 0.01 level (2-tailed)

### Demographic results of participants

The mean age of 213 samples was 37.31 ± 7.17 years. 75.1% were women, 51.4% were married, and 77.5% had academic education. Other demographic variables are shown in Table 4.

**Table 4:** Demographic characteristics of the study subjects (*n* = 213)

Variables		Frequency	
		Mean + SD	
Age (year)		37.31 + 7.17	
		Number	Percent
Education	Elementary school	3	1.4
	high school	45	21.1
	Academic	165	77.5
Sex	Female	160	75.1
	Male	53	24.9
Marital status	Single	104	48.6
	Married	109	51.4
Job	Full time	78	36.6
	Part time	15	7
	College student	54	25.4
	Unemployment	66	31

## Discussion

The availability of particular, legitimate, and trustworthy data collection instruments is one of the most fundamental factors in performing any research. Then, the first step in promoting media health literacy is to measure it. Therefore, the present study was conducted with the aim of developing and psychometric a tool to assess the media health literacy of Iranian adults. MeHLit is the first tool to assess media health literacy in adults. MeHLit is a valid and reliable assessment tool to measure media health literacy regarding individuals’ skills to assess health-related messages which will be received by noticing the goal of, type of content, meaning, appearance, and audience of a message. Each of the two concepts of health literacy [36] and media literacy [6, 37–39] have been measured separately through different instruments. Based on our knowledge, so far no specific tool has been found to measure media literacy and health. MeHLit was designed by deductive-inductive approach [40]. The proufound literature review demonstrated the concept of media health literacy is unique in that it provides more than just health advice considering information conveyed by the media. However, exposure to implicit or explicit mass media content smartly for the health care system is vital. Accordingly, the EFA indicated five factors which all of which were similar to concepts of the framework. This valid and reliable tool is a reflection of the intertwining of two main concepts, media literacy, and health. MeHLit is able to measure 5 main skills of an individual in relation to encountering health-related messages in the media.

The face and content validity of MeHLit was confirmed qualitatively and quantitatively. The proper validity of a questionnaire usually refers to the vision of the target group about face validity, suitability, attractiveness, comprehensibility, cultural and social appropriateness, logical sequence of the elements, and the completeness of the instrument [40]. In qualitative face validity, 3 items were deleted due to vague and duplication. In the quantitative face validity assessment, the impact score of all items was higher than 1.5 and was shown to be acceptable. Content validity of MeHLit was also confirmed by CVR = 0.87(0.8) and CVI = 0.93(0.8–1), respectively. It shows that MeHLit has an appropriate sample of items for assessing Media Health Literacy.

Results of EFA showed “Goal appraisal skill”; with 7 items explaining the highest variance (35.65%) and as the first domain of the MeHLit refers to skill, which had the greatest contribution in explaining media health literacy, and understanding of the purpose of the message sender. The current literature focused on media health literacy revealed that there is a relationship between rumor, infodemic management, and media literacy in the health care system [41, 42]. Accordingly, rumors will be three times more share than verified stories [43]. Therefore, the skill of appraising any distributed and shared message on health issues is vital. Content appraisal skill; with 5 items and 8.55% of the variance was the second domain of the MeHLit which assess the ability of a message receiver for content analysis. This skill determines whether or not a person is able to assess the accuracy of the message and able to critique that. The third domain of MeHLit refers to the “Implicit meaning appraisal skill” with 4 items explaining 6.10% of variance focuses on the audience’s ability to pay attention to the hidden message of each message they received and consider the pros and cons of sharing it. Several studies confirm that Implicit and explicit health messages are increasingly prevalent in media advertising [44, 45]. However, considering media health literacy as the noticeable approach in order to address this issue is essential. Visual Comprehension skill was extracted as the fourth domain of MeHLit with 3 items and 5.43% of the variance. This dimension refers to the type of design and visual factors in the attractiveness of a message. By understanding the validity of a message’s visual content, the user can proceed and criticize the message’s visual manipulation [46, 47] The results of the study by Samantha Golding et al. (2020) showed that visual literacy training can increase the understanding of educational concept [48]. Finally, “Audience appraisal skill”; with 2 items explaining the lowest variance (4.52%), and the last domain of the MeHLit refers to skill, which had the least contribution in explaining media health literacy. This domain assesses the audience’s skill for understanding who is the main audience of this message?, Or for whom this message is designed and appropriate.

Despite these limitations, this research can be seen as a first step towards integrating two lines of research, media literacy and health, which to our knowledge, have been directly linked. Therefore, the present research contributes to a growing body of evidence suggesting that a valid and reliable instrument can assess media health literacy, which focuses on necessary skills for media health literacy. Nonetheless, the discussion could have been richer and more multifaceted if there were similarly studied to compare findings. The research team of this study believes that the dominancy of the female participants in terms of number, the use of online social networks to collect data, and the focus on the young age group with high literacy may affect the tool’s evaluation power. It seems that this limitation is due to the convenient sampling method of this study, which was common in the conditions of the Covid-19 pandemic. However, to have a better understanding of the sensitivity and accuracy of the MeHLit, it is necessary for this tool to be tested in several interventional studies. It is recommended also, that this instrument be tested on people with different levels of education.

## Conclusion

MeHLit is a 21-item valid CVR = 0.87 (0.8–1) and CVI = 0.93 (0.82–1) and reliable (Cronbach’s α = 0.91) questionnaire with five domains including; Goal appraisal skill”; “Content appraisal skill” “Implicit meaning appraisal skill”; “Visual Comprehension skill”; “Audience appraisal skill”; which predict 60.25% of the variance.

## Data Availability

The datasets generated and/or analyzed during the current study are not publicly available due to information about the personal issues of the participants in this study contradicts the confidentiality of information but are available from the corresponding author on reasonable request.

## Abbreviation

MeHLit: Media Health Literacy
